# Pterygium: what about point of care biomarkers?

**DOI:** 10.1007/s00347-021-01414-4

**Published:** 2021-05-31

**Authors:** Alexander C. Rokohl, Ludwig M. Heindl, Claus Cursiefen

**Affiliations:** 1 Zentrum für Augenheilkunde, Universitätsklinikum Köln, Kerpener Str. 62, 50924 Köln, Deutschland; 2 Zentrum für Molekulare Medizin Köln (ZMMK), Universität zu Köln, Köln, Deutschland

## Erwiderung

Zum Leserbrief von Gonçalves dos Santos Martins T, Anschütz A, Kaczmarczyk C (2021) Biomarker bei Pterygium. Ophthalmologe. 10.1007/s00347-021-01413-5.

## Originalbeitrag

Rokohl AC, Heindl LM, Cursiefen C (2021) Pterygium: Pathogenese, Diagnose und Therapie. Ophthalmologe. 10.1007/s00347-021-01366-9.

Mit großem Interesse haben wir den Beitrag von Thiago Gonçalves dos Santos Martins, Andreas Anschütz und Carmen Kaczmarczyk über Biomarker in der Pathogenese und der Therapie des Pterygiums gelesen. Wir danken den Autoren für diese wichtigen Ergänzungen zu diesem Thema.

Verschiedene Studien haben bereits mögliche molekulare Mechanismen der Pterygiumentstehung untersucht [1–6]. Diese molekularen Mechanismen umfassen, teilweise durch UV-Strahlung oder virale Infektionen initiiert, unter anderem oxidativen Stress, Modulation der Extrazellularmatrix, Veränderung der Expression von apoptotischen Proteinen und Tumorsuppressorgenen, Verlust der Heterozygose, DNA-Methylierung, erhöhte Expression von Entzündungsmediatoren, gesteigerte (Lymph‑)Angiogenese und Veränderungen des Cholesterinstoffwechsels [1–5]. Diese Studien legen zudem nahe, dass insbesondere Matrixmetalloproteinasen, Wachstumsfaktoren und Interleukine zu einer vermehrten (Lymph‑)Angiogenese, gesteigerter Entzündungsreaktion sowie zu einer pathologisch gesteigerten Proliferation von Fibroblasten zu führen scheinen [1–5]. Die KollegInnen aus Brasilien, Deutschland und England haben völlig recht, dass auch der durch das humane Papillomavirus (HPV) verursachte Mechanismus einer über E6- und E7-Faktoren vermittelten Inaktivierung von p53 wie auch der NOD-ähnliche Pyrin3(NLRP3)/Caspase-1-Signalweg bei der Entwicklung eines Pterygiums eine herausragende Rolle einnehmen [1, 2].

Die Summe all dieser molekularen Mechanismen ist für das Verständnis der Ätiopathogenese des Pterygiums essenziell und spielt insbesondere in der (Rezidiv‑)Prophylaxe eine wichtige Rolle [1–5]. Eine konsequente Vermeidung einer UV-Exposition, z. B. durch das Tragen einer UV-schützenden Sonnenbrille oder in Zukunft ggf. auch UV-blockierender Kontaktlinsen, ist zur Prophylaxe beispielsweise unerlässlich [1–4, 7]. Die Anwendung von Mitomycin C hemmt die Expression von Entzündungsmediatoren wie TGF-β1, VEGF und IL‑6. Daher können zur Rezidivprophylaxe Antimetaboliten wie Mitomycin C sowohl intraoperativ als auch postoperativ verabreicht werden [1–4]. Die postoperative Reduktion proinflammatorischer Botenstoffe wie Interleukin‑1 dient ebenfalls der Senkung des Rezidivrisikos [1, 4, 7–10]. Dabei können sowohl Cyclosporin A oder Kortikosteroide, möglichst ohne Konservierungsmittel, für einige Wochen oder Monate topisch angewendet werden [1–4]. Eine antiangiogene adjuvante Therapie mit monoklonalen Antikörpern gegen den vaskulären endothelialen Wachstumsfaktor (beispielsweise Bevacizumab) zielt auf die Reduktion von proangiogenen Faktoren wie „vascular endothelial growth factor“ (VEGF) und „connective tissue growth factor“ (CTGF) ab und kann so ebenfalls zur Rezidivreduktion führen [1–4, 11].

Während die Entschlüsselung einiger molekularer Mechanismen das Verständnis der Ätiopathogenese und damit auch die Rezidivprophylaxe verbessert hat, fehlen bis heute Point-of-Care-Tests für diese Biomarker in der klinischen Routine. Diese Point-of-Care-Tests könnten beispielsweise dazu dienen, erhöhte Konzentrationen von Interleukinen oder Metalloproteinasen z. B. im Tränenfilm zu detektieren. Einerseits könnte so unter Umständen das Ansprechen auf eine konservative antiinflammatorische Therapie zur Verhinderung einer Progression monitort werden. Andererseits könnten ggf. Patienten, die ein erhöhtes Risiko für eine Progression oder für ein Rezidiv haben, identifiziert werden. Diese Erkenntnisse könnten die Entscheidung für eine frühzeitigere chirurgische Intervention oder für die Anwendung von Mitomycin C signifikant beeinflussen. Nun ist es eine wichtige Forschungsaufgabe, entsprechende Point-of-Care-Tests für relevante Biomarker zu etablieren und den Nutzen in klinischen Studien zu prüfen [12].
